# Investigating the effects of tracheostomy cuff deflation timing in patients requiring prolonged mechanical ventilation – An observational study

**DOI:** 10.1371/journal.pone.0355055

**Published:** 2026-08-07

**Authors:** Adam Harriman, Dhruv Parekh, William Tunnicliffe, Catherine Snelson, James Hodson, Ruth Capewell, Molly Metcalf, Camilla Dawson, Jonathan Weblin

**Affiliations:** 1 Therapy Services, University Hospitals Birmingham NHS Foundation Trust, Birmingham, United Kingdom; 2 Birmingham Acute Care Research Group, University of Birmingham Institute of Inflammation and Ageing, Birmingham, United Kingdom; 3 Intensive Care Medicine, University Hospitals Birmingham NHS Foundation Trust, Birmingham, United Kingdom; 4 Research Development and Innovation, University Hospitals Birmingham NHS Foundation Trust, Birmingham, United Kingdom; Ataturk University Faculty of Medicine, TÜRKIYE

## Abstract

**Introduction:**

Tracheostomy insertion is common in patients requiring prolonged mechanical ventilation (MV), with cuff deflation and voice restoration a critical step in the weaning process. However, the optimal timing for cuff deflation and its impact on clinical outcomes remain unclear. This study aims to examine local tracheostomy cuff deflation practices and assess associations with outcomes in patients requiring prolonged MV.

**Methods:**

A retrospective study was conducted at a large UK ICU between November 2021 and October 2023, which included patients without significant neurological injury requiring tracheostomy insertion for prolonged MV (defined as WIND 3). Data were extracted from electronic health records for patient demographics and outcomes including weaning milestones; physical, cognitive, and psychological outcomes; ICU and hospital length of stay (LOS); ventilator-acquired pneumonia (VAP); and mortality. These were compared across subgroups defined based on the time from tracheostomy to cuff deflation, namely 0–5, 6–10, and >10 days; patients who died prior to achieving this were included in the latter subgroup.

**Results:**

The 157 included patients comprised those with tracheostomy to cuff deflation of 0–5 days (N = 58), 6–10 days (N = 54) and >10 days/death (N = 45). Patient characteristics at ICU admission were not found to differ significantly between these groups. Delayed cuff deflation was associated with prolonged MV (p < 0.001); delayed weaning milestones, including decannulation (p < 0.001); higher rates of VAP (p = 0.010); increased ICU LOS (p < 0.001) and mortality (p < 0.001); and greater levels of anxiety (p = 0.040) and psychological distress (p = 0.046) at ICU discharge.

**Conclusion:**

Delayed tracheostomy cuff deflation maybe associated with inferior clinical and patient outcomes. Further research examining the relationship between cuff deflation timing and clinical outcomes is warranted.

## Introduction

Tracheostomy insertion is performed in 10–15% of patients admitted to intensive care units (ICUs) [1]. One indication for tracheostomy insertion is to aid weaning in patients requiring prolonged mechanical ventilation (MV). Around 15% of intubated patients require ongoing MV beyond 7 days [2], with around 4% requiring MV beyond 60 days [3]. Tracheostomy insertion generally allows patients to be managed with less sedation, facilitating greater wakefulness, which promotes their ability to engage with the weaning process and rehabilitation, and improves their comfort and safety [4]. Patients requiring tracheostomies generally have a longer ICU length of stay (LOS), greater comorbidity, and higher mortality rates [5]. They may also experience physical, cognitive and psychological morbidity, including anxiety, depression, post-traumatic stress [6], temporary or permanent loss of speech [7], difficulty swallowing [8], pain, increased work of breathing, tracheal trauma and bleeding, and tracheal stenosis [9]. Consequently, it would be expected that earlier tracheostomy removal could result in improved outcomes in patients requiring prolonged MV. However, whilst there is an extensive evidence base concerning the indications for, timing of, and techniques of tracheostomy insertion and their subsequent removal, little attention has been given to optimal management between the time of insertion in ICU and subsequent ICU discharge [10]. Similarly, evidence underpinning the most effective techniques that might accelerate the processes of weaning tracheostomy patients from prolonged MV is limited [11].

Weaning ventilatory support in tracheostomised patients generally follows a linear, stepwise process, although clinical practice varies. Generally, weaning ventilatory support starts with restoring spontaneous breathing effort, followed by a sequential reduction in inspiratory support towards continuous positive airway pressure (CPAP), then the introduction of supplemental oxygen via tracheostomy mask, prior to tracheostomy cuff deflation as a prelude to decannulation [12]. Delays in tracheostomy cuff deflation in the weaning process prevents vocalisation, and could impact swallow, cough effectiveness and protective laryngeal competence as a result of deconditioning [13]. Structured, protocolised weaning has been associated with a reduction in ICU LOS and an increase in ventilator-free days [14]. However, there is limited robust evidence comparing the effectiveness of different weaning approaches and techniques in patients with tracheostomies. The restoration of normal anatomical airflow, through tracheostomy cuff deflation and speaking valve use, is accepted as standard practice with tracheostomy patients. However, its role during the earlier phases of MV is less well established in both clinical practice and the literature. The safety and feasibility of cuff deflation within 24 hours of tracheostomy insertion has been demonstrated [15]. Despite this, there is little evidence examining the benefits on secondary outcomes such as ventilator-free days and LOS, with the limited current research reporting inconsistent results. One randomised controlled trial found early cuff deflation to be associated with reductions in the time to successful tracheostomy removal, as well as ICU and hospital LOS [16]. Freeman-Sanderson also reported that early cuff deflation facilitated earlier restoration of phonation without an associated increase in adverse events. However, compared with standard care, earlier cuff deflation did not result in a significant difference in time to decannulation and or liberation from mechanical ventilation [13].

Here, we report outcomes for patients without neurological injury who underwent tracheostomy insertion on our ICU over a two-year period and were deemed ‘challenging to wean’. The aims of this service evaluation were three-fold. Firstly, to define current practice regarding the circumstance and timing of initial cuff deflation within ‘challenging to wean’ patients. Secondly, to explore patient and clinical factors related to the timing of cuff deflation. Thirdly, to explore associations between timing of cuff deflation and physical, cognitive and psychological outcomes, to inform the design of a potential future trial exploring early cuff deflation.

## Methods

### Setting

The study was undertaken in the ICU at the Queen Elizabeth Hospital Birmingham, United Kingdom. This 100-bed, single-site facility comprises four subspecialty units, caring for an extensive range of adult elective and emergency admissions. Each unit has twice-daily consultant-led medical ward rounds and access to a wide interdisciplinary team (IDT) including Physiotherapy, Occupational Therapy, Speech and Language Therapy (SLT) and Dietitians. An embedded Critical Care Physiotherapy Rehabilitation Team (CCPRT) delivers enhanced rehabilitation to patients without significant neurological or traumatic injury or cardiothoracic transplantation, who have an anticipated duration of MV ≥ 4 days, in line with national recommendations [17,18]. A dedicated ICU SLT team deliver interventions aimed to restore voice and swallow function in tracheostomised patients. In addition, a weekly consultant-led, IDT, ‘long-stay ward round’ (LSWR) for patients with an ICU LOS of ≥14 days provides advice on weaning and tracheostomy practice, including timing of cuff deflation. However, neither the process of ventilator weaning nor the timing of tracheostomy cuff deflation are protocolised; therefore, practice varies between individual clinicians and subspecialties. Generally, cuff deflation would be attempted when a tracheostomised patient is off sedation (awake and alert); sufficiently medically stable to engage in active rehabilitation and ventilator weaning; and attempting to verbally communicate. For patients requiring prolonged MV, the LSWR generally advocates a weaning strategy of gradual reduction of inspiratory support during the day, whilst maintaining elevated overnight inspiratory support to ‘rest’ patients. When daytime liberation to supplementary oxygenation alone via tracheostomy mask has been achieved, the duration of overnight support is gradually reduced until full liberation is achieved.

### Technique for delivering ventilatory support with a deflated tracheostomy cuff

The ‘routine’ ventilator used at the study site cannot tolerate cuff deflation while delivering positive pressure, as the leak imposed by cuff deflation disrupts ventilation. To facilitate cuff deflation, patients are transferred onto a turbine-driven ‘weaning’ ventilator (Nihon Kohden NKV-330), which can compensate for leaks of up to 200L/minute while still delivering pressure support ventilation (PSV) up to approximately 16 cmH_2_O via a humidified circuit. This may enable earlier cuff deflation during the weaning process, with the resulting airflow through the larynx promoting phonation. An in-line, closed position biased speaking valve can be utilised to improve phonation when lower levels of PSV or no PSV is being delivered. The decision of when to transfer a patient to the ‘weaning’ ventilator (NKV-330) is made by the treating clinician.

### Data collection

Patients admitted to the ICU between the 1^st^ of November 2021 and 31^st^ October 2023 were retrospectively identified from electronic health records system (EHR). From these, patients who required at least one day of MV, had a tracheostomy inserted, and were deemed ‘challenging to wean’ were identified. Patients were excluded if tracheostomy insertion was due to severe neurological impairment; Ear, Nose and Throat (ENT) surgery; or cardiac or lung transplantation. ‘Challenging to wean’ was defined as Weaning According to a New Definition group 3 (WIND 3) [19], specifically where liberation from MV was not achieved within 7 days of the first separation attempt. The resulting cohort was also cross-referenced against the prospective database maintained by the CCRT, to ensure complete case ascertainment. Patients not under the care of the CCRT were then excluded, due to differences in standard rehabilitation practice. For the resulting cohort, demographic data, weaning milestones and MV/patient outcomes were retrospectively extracted from the EHR.

Patients’ physical function was assessed at ICU and hospital discharge using the Manchester Mobility Score (MMS). The severity of ICU-acquired weakness (ICU-AW) at ICU discharge was assessed using the Medical Research Council Sum Score for Muscle Strength Evaluation (MRC-SS) and classified as: none (MRC-SS ≥ 48), significant (MRC-SS: 36–47) or severe (MRC-SS < 36). Assessments of delirium were performed daily in ICU using the Confusion Assessment Method (CAM); patients were then classified based on any identification of delirium during the ICU stay, or at the point of ICU discharge. The Intensive Care Psychological Assessment tool (IPAT) was performed on the day of ICU discharge, or on the nearest weekday if ICU discharge fell at the weekend, with IPAT≥7 indicating risk of psychological dysfunction. Hospital Anxiety and Depression Scale (HADS) was collected at the same time points, and used to measure anxiety (HADS-A) and depression (HADS-D), with scores subsequently grouped into a four-point ordinal scale of: normal (HADS: 0–7), mild [8–10], moderate [11–15] or severe (> [15]); HADS≥8 was deemed to represent clinically significant anxiety/depression.

Where patients achieved a weaning milestone, the date that this first occurred was extracted, and the number of days from tracheostomy insertion was calculated. These milestones comprised: cessation of sedation, first cuff deflation attempt, respiratory support of supplemental oxygenation via tracheostomy mask (TM) alone for the first time, achieving 24 hours of TM, use of a speaking valve, and decannulation. Milestones in the progression of reduction in PSV were also recorded, specifically the use of spontaneous ventilation modes, transfer to a ‘weaning’ ventilator; and the first CPAP trial. The level of PS at first cuff deflation was classified as TM, CPAP, or PSV of 1–5, 6–10 or >10 cmH_2_O. The number of days from initiation of MV to the first spontaneous breath and total MV days were also recorded.

Patient outcomes included mortality in ICU, hospital and within 3-months post-discharge; and the ICU and hospital LOS (with the latter defined as time from ICU admission to hospital discharge). ICU and hospital LOS calculations excluded those who died prior to achieving successful ICU or hospital discharge, and those transferred to another hospital prior to leaving ICU. Incidence of ventilator-acquired pneumonia (VAP) was identified, defined as a consultant-led decision to commence antibiotics for suspected VAP. Each patient’s ongoing rehabilitation requirement was quantified based on their hospital discharge destination, which was classified as: home without rehabilitation, home with ongoing rehabilitation, or an inpatient rehabilitation facility.

### Statistical methods

The time from tracheostomy to cuff deflation was summarised for the cohort using a Kaplan-Meier curve, which censored patients who died before cuff deflation at the time of death. Patients were then divided into three subgroups of approximately equal sample size based on time to cuff deflation. Patients who died prior to achieving cuff deflation were reviewed, to decide on the most appropriate group assignment. Patient characteristics and outcomes were then compared across the three subgroups using Jonckheere-Terpstra tests for ordinal or continuous variables. Binary variables analysed using Mann-Whitney U tests, with the ordinal time to cuff deflation category treated as the dependent variable.

All analyses were performed using IBM SPSS v29 (IBM Corp. Armonk, NY), with p < 0.05 deemed to be indicative of statistical significance throughout. Continuous variables are reported as *mean ± standard deviation* where approximately normally distributed, or as *median (interquartile range; IQR)* otherwise. Cases with missing data or where a factor was not applicable were excluded from the analysis of the affected factor; the numbers of cases included in each analysis are reported in the tables.

### Registration

This study was registered with the site’s Research and Development team through the Clinical Audit Registration & Management System (CARMS-18704) and deemed to be a retrospective service evaluation; hence, formal ethical approval was not sought.

## Results

### Derivation of Study Cohort

**Fig 1:**
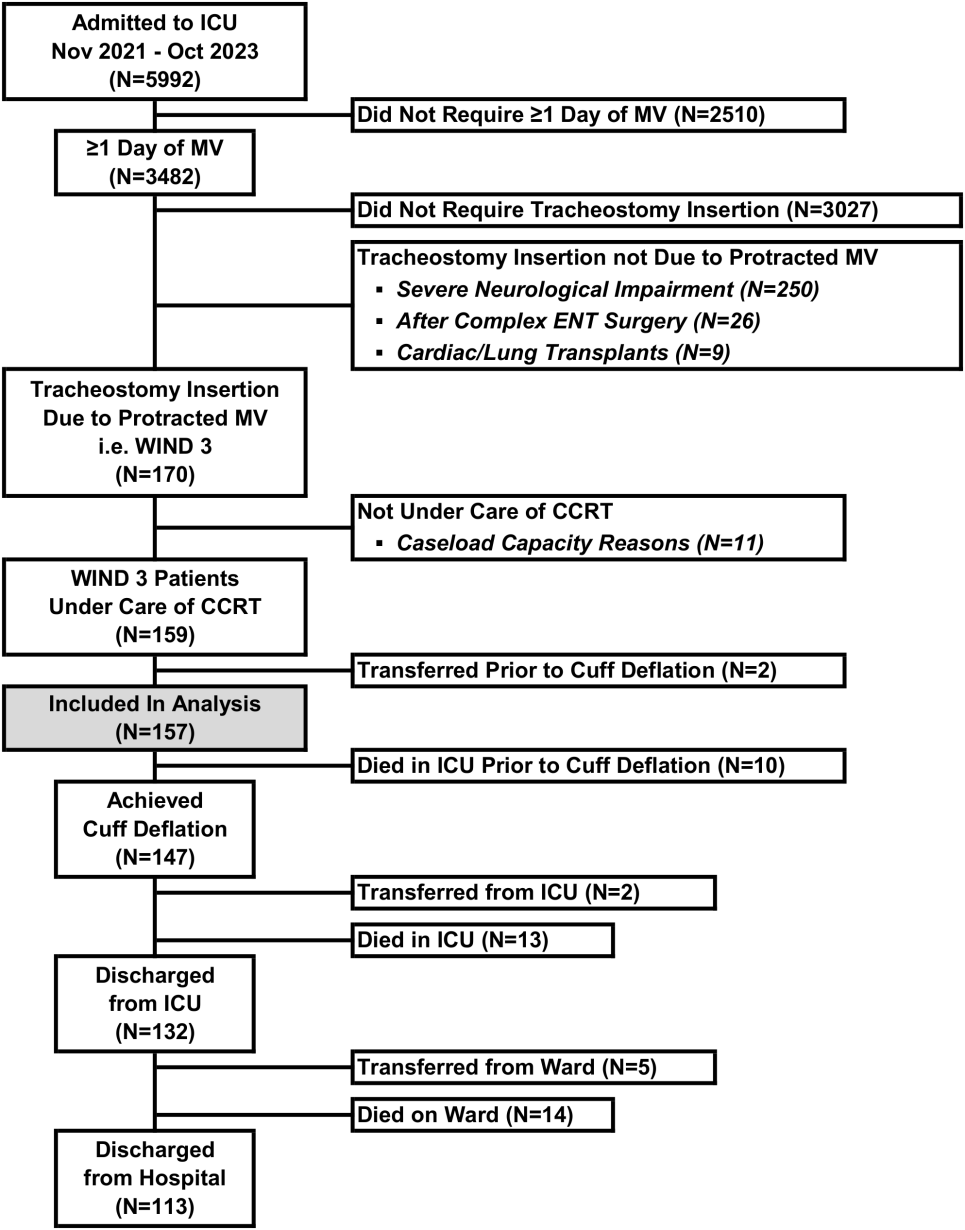
Study flowchart. Abbreviations: CCRT: Critical Care Rehabilitation Team, ENT: Ear Nose and Throat, MV: Mechanical Ventilation, ICU: Intensive Care Unit, WIND 3: Weaning According to a New Definition – Group 3.

A total of 5992 patients were admitted to the ICU, of whom 3482 (58.1%) required at least one day of MV (median ICU LOS: 3 days, IQR: 2–5) and 1418 (23.7%) required ≥4 days of MV (median ICU LOS: 14 days, IQR: 8–24). Tracheostomy insertion was performed in 455 patients, of which 170 were for the provision of protracted MV due to respiratory failure; hence, were classified as WIND 3. Of these, 11 patients were excluded as they were not placed under the supervision of the CCRT, due to the team having insufficient capacity at the time of the referral. A further 2 patients were excluded from the analysis as they were transferred from the ICU to another hospital prior to cuff deflation; hence, the timing of cuff deflation was not recorded. The remaining 157 WIND 3 patients were included in the study (Fig 1).

### Cohort Characteristics

The mean age of the cohort at ICU admission was 57.6 ± 14.8 years and 67% were male; most patients had percutaneous tracheostomies (75%; Table 1). Five (3%) patients died whilst still on MV, with the remainder having a median MV duration of 29 days (IQR: 19–38). A total of 23 (15%) patients died in the ICU, with a further 2 (1%) transferred from the ICU to another hospital; the 132 who were discharged from ICU had a median ICU LOS of 34 days (IQR: 24–46). Psychological and cognitive morbidity were common in the ICU, with 77% of patients having evidence of delirium, and over half of those who survived to ICU discharge and underwent an assessment at this time demonstrating clinically significant anxiety (53%) or depression (52%) or being at risk of psychological distress (67%). Where patients survived ICU, ICU-AW was common, being significant to severe in 55% of patients, with 43% being unable to walk (MMS < 6) at ICU discharge (Table 1).

**Table 1:** Cohort characteristics by time from tracheostomy insertion to cuff deflation.

			Tracheostomy to Cuff Deflation			p- Value
	N	Whole Cohort	*0-5 Days*	*6-10 Days*	*>10 Days/ Death*	
***Patient Characteristics at ICU Admission***						
Age (Years)	157	57.6 ± 14.8	58.0 ± 15.3	57.0 ± 14.0	57.7 ± 15.5	0.901
Male Sex	157	105 (67%)	40 (69%)	35 (65%)	30 (67%)	0.774
BMI (kg/m^2^)	157	27.5 ± 6.4	26.9 ± 6.3	28.1 ± 6.9	27.5 ± 6.0	0.403
Charlson Comorbidity Index	157	3 (2 –5)	3 (2 –5)	3 (1 –4)	3 (2 –5)	0.793
APACHE II Probability (%)	157	18 (8-29)	19 (8-31)	15 (9-28)	19 (8-26)	0.390
Tracheostomy Type	157					0.929
*Percutaneous*		118 (75%)	45 (78%)	38 (70%)	35 (78%)	
*Surgical*		39 (25%)	13 (22%)	16 (30%)	10 (22%)	
***MV/ICU Outcomes***						
MV to First Spont. Breath (Days)	155	4 (2 –6)	4 (2 –6)	4 (2 –6)	3 (2 –7)	0.874
Duration of MV (Days) ^**a**^	152	29 (19-38)	20 (13 –29)	30 (20 –36)	36 (32-45)	**<0.001**
ICU Admission to Tracheostomy (Days)	157	11 (8 –15)	11 (7 –18)	11 (8 –15)	12 (9 –17)	0.389
Ventilator-Acquired Pneumonia	157	83 (53%)	23 (40%)	31 (57%)	29 (64%)	**0.010**
Died in ICU	157	23 (15%)	4 (7%)	3 (6%)	16 (36%)	**<0.001**
ICU Length of Stay (Days) ^**b**^	132	34 (24-46)	25 (17-34)	33 (25-45)	48 (39-57)	**<0.001**
MRC-SS at ICU Discharge ^**bc**^	117	46 (36-50)	48 (40-50)	46 (35-50)	44 (35-49)	0.180 ^**c**^
*No ICU-AW*		53 (45%)	22 (52%)	18 (38%)	13 (46%)	
*Significant ICU-AW*		38 (32%)	15 (36%)	17 (36%)	6 (21%)	
*Severe ICU-AW*		26 (22%)	5 (12%)	12 (26%)	9 (32%)	
MMS at ICU Discharge ^**b**^	131					**0.013** ^**d**^
*3*		16 (12%)	4 (8%)	7 (14%)	5 (18%)	
*4*		31 (24%)	11 (21%)	9 (18%)	11 (39%)	
*5*		9 (7%)	4 (8%)	5 (10%)	0 (0%)	
*6*		39 (30%)	14 (27%)	16 (31%)	9 (32%)	
*7*		36 (27%)	19 (37%)	14 (27%)	3 (11%)	
Delirium in ICU	152	117 (77%)	44 (76%)	41 (77%)	32 (78%)	0.792
Delirium at ICU Discharge ^**b**^	118	25 (21%)	9 (19%)	12 (27%)	4 (16%)	0.949
HADS-A at ICU Discharge ^**bc**^	89	8 (4 –11)	7 (3 –9)	8 (6 –12)	8 (4 –14)	**0.040** ^**c**^
*Normal*		42 (47%)	20 (56%)	15 (42%)	7 (41%)	
*Mild Anxiety*		24 (27%)	12 (33%)	10 (28%)	2 (12%)	
*Moderate Anxiety*		17 (19%)	2 (6%)	10 (28%)	5 (29%)	
*Severe Anxiety*		6 (7%)	2 (6%)	1 (3%)	3 (18%)	
HADS-D at ICU Discharge ^**bc**^	89	8 (4 –12)	6 (3 –10)	8 (5 –12)	10 (4 –15)	0.056 ^**c**^
*Normal*		43 (48%)	21 (58%)	15 (42%)	7 (41%)	
*Mild Depression*		22 (25%)	8 (22%)	11 (31%)	3 (18%)	
*Moderate Depression*		15 (17%)	5 (14%)	5 (14%)	5 (29%)	
*Severe Depression*		9 (10%)	2 (6%)	5 (14%)	2 (12%)	
IPAT at ICU Discharge ^**bc**^	89	8 (6 –12)	8 (4 –11)	9 (7 –12)	12 (6 –15)	**0.046** ^**c**^
*Psychological Distress*		60 (67%)	21 (58%)	27 (75%)	12 (71%)	

*Analyses are based on N = 157, after excluding those with missing data, unless stated otherwise. Continuous variables are reported as ‘mean ± standard deviation’ or ‘median (interquartile range)’, as applicable, with p-values from Jonckheere-Terpstra tests. Categorical variables are reported as ’N (column %)’, with p-values from Mann-Whitney U tests (i.e., with the tracheostomy to cuff deflation group as the dependent variable), unless stated otherwise. Bold p-values are significant at p < 0.05.* ^*a*^ *Excludes patients who died on MV (N = 5).* ^*b*^ *Excludes patients who died in the ICU (N = 23) or were transferred from the ICU (N = 2).* ^**c**^ *The primary analysis is on the MRC-SS/MMS/IPAT score, for which the medians (interquartile ranges) are reported, and compared between groups using Jonckheere-Terpstra tests; the proportion of patients within subgroups are additionally reported, to simplify interpretation.* ^*d*^ *p-Value from Jonckheere-Terpstra test, as the factor is ordinal. Abbreviations: BMI: Body Mass Index, HADS-A(-D) Hospital Anxiety and Depression Scale-Anxiety (-Depression) Component, ICU: Intensive Care Unit, ICU-AW: ICU-Acquired Weakness, IPAT: Intensive Care Psychological Assessment Tool, MMS: Manchester Mobility Score, MRC-SS: Medical Research Council Sum Score for Muscle Strength Evaluation, MV: Mechanical Ventilation, Spont.: Spontaneous.*

A further 14 (9%) patients died in hospital after ICU discharge, with 5 (3%) transferred to another hospital. The remaining 113 (72%) who survived to hospital discharge had a median hospital LOS of 50 days (IQR: 38–74), with the majority having MMS ≥ 6 (88%) and being discharged home, either with (36%) or without (45%) ongoing rehabilitation (Table 2).

**Table 2:** Hospital outcomes by time from tracheostomy insertion to cuff deflation.

			Tracheostomy to Cuff Deflation			p- Value
	N	Whole Cohort	*0-5 Days*	*6-10 Days*	*>10 Days/ Death*	
Died in Hospital	157	37 (24%)	9 (16%)	8 (15%)	20 (44%)	**0.001**
Hospital Length of Stay (Days) ^**a**^	113	50 (38-74)	46 (30-74)	48 (38-64)	64 (49-89)	**0.005**
MMS at Hospital Discharge ^**a**^	113					0.053 ^**b**^
*2*		1 (1%)	1 (2%)	0 (0%)	0 (0%)	
*3*		4 (4%)	0 (0%)	2 (4%)	2 (10%)	
*4*		6 (5%)	2 (4%)	4 (9%)	0 (0%)	
*5*		2 (2%)	1 (2%)	1 (2%)	0 (0%)	
*6*		26 (23%)	8 (17%)	10 (22%)	8 (40%)	
*7*		74 (65%)	36 (75%)	28 (62%)	10 (50%)	
Discharge Destination ^**a**^	113					**0.039** ^**b**^
*Home (No Rehabilitation)*		51 (45%)	27 (56%)	18 (40%)	6 (30%)	
*Home (With Rehabilitation)*		41 (36%)	14 (29%)	18 (40%)	9 (45%)	
*Inpatient Rehabilitation*		21 (19%)	7 (15%)	9 (20%)	5 (25%)	
Died 3 Months Post-Discharge ^**c**^	118	6 (5%)	2 (4%)	0 (0%)	4 (17%)	0.120

*Analyses are based on N = 157, after excluding those with missing data, unless stated otherwise. Continuous variables are reported as ‘median (interquartile range)’, with p-values from Jonckheere-Terpstra tests. Categorical variables are reported as ‘N (column %)’, with p-values from Mann-Whitney U tests (i.e., with the tracheostomy to cuff deflation group as the dependent variable), unless stated otherwise. Bold p-values are significant at p < 0.05.* ^***a***^ *Excludes patients who died in hospital (N = 37) or were transferred from hospital (N = 7).* ^***b***^ *p-Value from Jonckheere-Terpstra test, as the factor is ordinal.* ^***c***^ *Excludes patients who died in hospital (N = 37) or were transferred from the ICU (N = 2). Abbreviations: MMS: Manchester Mobility Score.*

### Timing of cuff deflation

**Fig 2:**
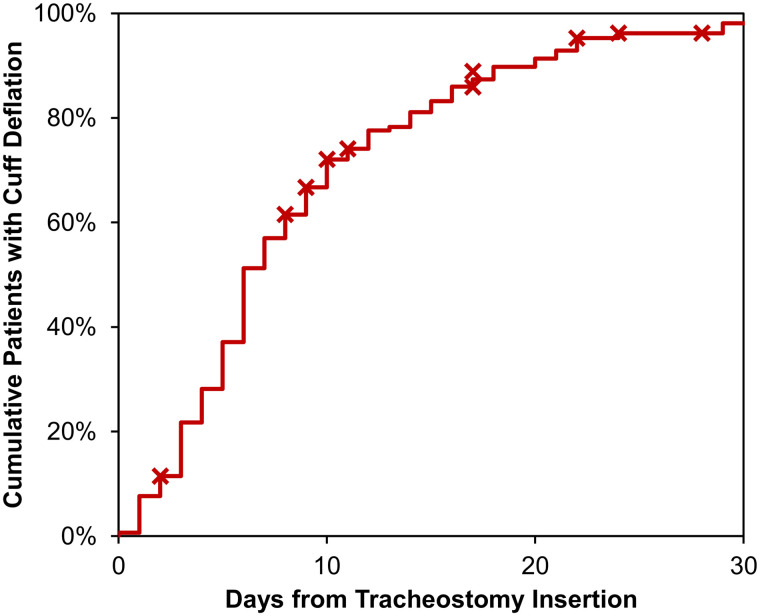
Kaplan-Meier curve of time to cuff deflation. Crosses represent patients who were censored at death prior to cuff deflation (N = 10); two patients died on day 17, hence points are plotted with jitter. One patient who died had a tracheostomy placed prior to ICU admission on an unknown date; for this patient, the date of ICU admission was used as a surrogate of the date of tracheostomy insertion. The x-axis is truncated at 30 days; one patient had cuff deflation beyond this (47 days from tracheostomy).

**Fig 3:**
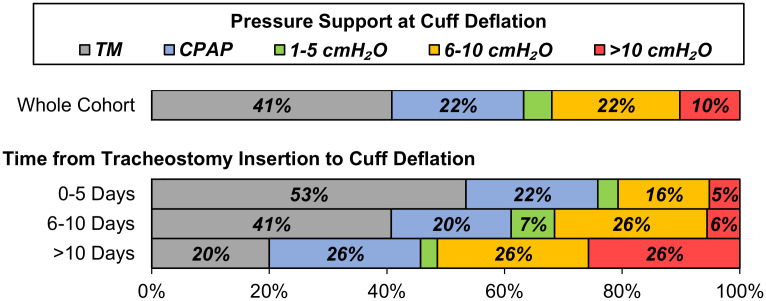
Levels of pressure support at cuff deflation. Results are based on the N = 147 patients that achieved cuff deflation.

Cuff deflation was not achieved in 10 (6%) patients. Of these, 2 died prior to the cessation of sedation and 8 died in the ICU between 8–28 days after tracheostomy insertion (median: 14 days). To account for these deaths, the average time from tracheostomy insertion to cuff deflation was estimated using a Kaplan-Meier approach (Fig 2), which returned a median of 6 days (IQR: 4–12). The longest observed time to cuff deflation was 47 days, due to a complete bilateral vocal cord palsy. At the point of cuff deflation, 41% of patients were using a TM, 22% were on CPAP, with the remaining 37% requiring PS (Fig 3).

### Associations with time to cuff deflation

Patients were divided into three groups based on the time from tracheostomy insertion to cuff deflation, namely 0–5 days (N = 58), 6–10 days (N = 54) and either >10 days or death (N = 45). For the latter group, the decision to include patients that died before cuff deflation was since most of these deaths occurred after >10 days; hence, it was assumed that these patients would have had an extended time to cuff deflation, had they survived long enough for this to be achieved. Comparisons between the three groups found no significant associations between the time to cuff deflation and patient demographics, including time to tracheostomy insertion (Table 1). However, patients with extended times to cuff deflation tended to have significantly longer durations of MV (p < 0.001) and ICU LOS (p < 0.001) and were significantly more likely to develop VAP (p = 0.010) or to die in the ICU (p < 0.001). No significant association was observed between the time to cuff deflation and rates of delirium in ICU (p = 0.792). However, in patients who survived to ICU discharge and for whom data were recorded, levels of anxiety (HADS-A, p = 0.040) and psychological distress (IPAT, p = 0.046) at this time were found to increase significantly with the time to cuff deflation.

Of the weaning milestones considered, whilst there was a tendency for patients with longer times to cuff deflation to have longer times from tracheostomy to both achieving spontaneous ventilation and stopping sedation, neither of these comparisons reached statistical significance (both p = 0.059). However, all other milestones were found to take significantly longer to achieve in patients with extended times to cuff deflation, specifically the times from tracheostomy to the first CPAP trial, transfer to a weaning ventilator, use of TMs and speaking valves, and to reaching 24 hours of TM, and time to decannulation (all p < 0.001, Table 3). Extended times to cuff deflation were also associated with significantly greater levels of PS at this time (p < 0.001, Fig 3).

**Table 3:** Weaning milestones by time from tracheostomy insertion to cuff deflation.

			Tracheostomy to Cuff Deflation			p- Value
	N	Whole Cohort	*0-5 Days*	*6-10 Days*	*>10 Days/ Death*	
Tracheostomy to Spont. Vent. > 1 Day ^**a**^	156	36 (23%)	12 (21%)	8 (15%)	16 (36%)	0.059 ^**a**^
Sedation Stopped	157	155 (99%)	58 (100%)	54 (100%)	43 (96%)	0.083
*Days from ICU Admission* ^**b**^	155	12 (8 –17)	11 (8 –16)	11 (8 –16)	15 (11 –19)	0.094
*Days from Tracheostomy* ^**bc**^	155	0 (0-2)	0 (0-1)	0 (0-1)	1 (0-6)	0.059
*Pre-Tracheostomy*		23 (15%)	9 (16%)	8 (15%)	6 (14%)	
*Same Day as Tracheostomy*		61 (39%)	26 (45%)	20 (37%)	15 (35%)	
*Next Day*		31 (20%)	13 (22%)	14 (26%)	4 (9%)	
*>1 Day*		40 (26%)	10 (17%)	12 (22%)	18 (42%)	
CPAP Trial	157	144 (92%)	56 (97%)	51 (94%)	37 (82%)	**0.013**
*Days from Tracheostomy* ^**b**^	144	3 (1 –6)	2 (1 –3)	3 (2 –6)	8 (3 –14)	**<0.001**
Transferred to ‘Weaning’ Ventilator	157	113 (72%)	38 (66%)	42 (78%)	33 (73%)	0.319
*Days from Tracheostomy* ^**b**^	113	5 (3 –9)	3 (1 –4)	6 (4 –7)	11 (7 –16)	**<0.001**
Tracheostomy Mask	157	144 (92%)	58 (100%)	53 (98%)	33 (73%)	**<0.001**
*Days from Tracheostomy* ^**b**^	144	7 (3 –16)	4 (2 –6)	8 (5 –12)	19 (14 –23)	**<0.001**
Cuff Deflation	157	147 (94%)	58 (100%)	54 (100%)	35 (78%)	**<0.001**
*Days to ICU Discharge* ^**d**^	132	12 (7-24)	10 (6 –16)	13 (9 –24)	15 (10 –26)	**0.043**
Pressure Support at Cuff Deflation ^**b**^	147					**<0.001** ^**e**^
*Tracheostomy Mask*		60 (41%)	31 (53%)	22 (41%)	7 (20%)	
*CPAP*		33 (22%)	13 (22%)	11 (20%)	9 (26%)	
*1-5 cmH*_*2*_*O*		7 (5%)	2 (3%)	4 (7%)	1 (3%)	
*6-10 cmH*_*2*_*O*		32 (22%)	9 (16%)	14 (26%)	9 (26%)	
*>10 cmH*_*2*_*O*		15 (10%)	3 (5%)	3 (6%)	9 (26%)	
Speaking Valve	157	140 (89%)	55 (95%)	51 (94%)	34 (76%)	**0.003**
*Days from Tracheostomy* ^**b**^	140	8 (5 –13)	4 (3 –5)	8 (7 –10)	16 (15 –21)	**<0.001**
Reached 24 Hours of TM ^**f**^	144	139 (97%)	57 (98%)	52 (98%)	30 (91%)	0.112
*Days from Tracheostomy* ^**b**^	139	14 (8-25)	8 (5 –14)	12 (10 –20)	28 (24 –34)	**<0.001**
Decannulated	157	128 (82%)	51 (88%)	50 (93%)	27 (60%)	**<0.001**
*Days from Tracheostomy* ^**b**^	128	21 (11-31)	11 (7-29)	21 (13 –29)	30 (24 –37)	**<0.001**

*Analyses are based on N = 157, after excluding those with missing data, unless stated otherwise. Continuous variables are reported as ‘median (interquartile range)’, with p-values from Jonckheere-Terpstra tests. Categorical variables are reported as ’N (column %)’, with p-values from Mann-Whitney U tests (i.e., with the tracheostomy to cuff deflation group as the dependent variable), unless stated otherwise. Bold p-values are significant at p < 0.05.* ^***a***^ *Due to a preponderance of patients achieving the outcome within 1 day, data are reported as the proportion of patients taking >1 day to more clearly show the difference between groups; the p-value is from a Jonckheere-Terpstra test on the original data***.** ^***b***^ *The number of days to first achieving the milestone, in those patients where it was achieved.* ^***c***^ *Negative times were assigned to patients who stopped sedation prior to tracheostomy insertion; data are additionally reported as the proportions of patients who stopped sedation within various time intervals.* ^***d***^ *Days from cuff deflation to ICU discharge, in patients who achieved cuff deflation and were discharged alive from ICU.* ^***e***^ *p-Value from Jonckheere-Terpstra test, as the factor is ordinal.* ^***f***^ *In patients who used a tracheostomy mask. Abbreviations: CPAP: Continuous Positive Airway Pressure, TM: Tracheostomy Mask, Spont. Vent.: Spontaneous Ventilation.*

Longer times to cuff deflation were associated with significantly higher rates of in-hospital mortality (p = 0.001, Table 2), significantly longer hospital LOS (p = 0.005), and greater requirement for post-discharge rehabilitation (p = 0.039) in those surviving to hospital discharge

## Discussion

This study presents the current clinical practice at our institute in relation to the circumstance and timing of initial tracheostomy cuff deflation. To our knowledge, it is one of the first studies to comprehensively review physical, cognitive and psychological outcomes in tracheostomised, WIND 3 patients. We identified the timing of tracheostomy insertion was consistent with current evidence-based practice, and most patients were established on spontaneous ventilation modes and off sedation within 24 hours of tracheostomy insertion. A low mortality rate was also observed, which may indicate that there was appropriate patient selection for tracheostomy and that the timing and process of weaning were generally appropriate. Weaning generally followed a linear pattern with the gradual reduction in ventilatory support, but considerable variability in the time from tracheostomy insertion to cuff deflation was observed, with most patients having the cuff deflated towards the end of the weaning process. This may reflect the lack of national guidelines around timing of cuff deflation in tracheostomy patients and of high-quality evidence to support early cuff deflation.

The relationships between patient characteristics at ICU admission and time to cuff deflation were also explored, which identified no statistically significant associations. In addition, no significant associations between the timing of cuff deflation and the times from ICU admission to tracheostomy insertion, or from tracheostomy insertion to either spontaneous ventilation or sedation cessation were detected. This raises the hypothesis as to whether delayed cuff deflation might in part be causal to, rather than because of, the prolongation of weaning. As shown in Fig 3, tracheostomy cuff deflation was also demonstrated to be possible during elevated PSV, indicting that it may be feasible to deflate the tracheostomy cuff earlier in a patient’s weaning process.

We also reported the impact of time to cuff deflation on the occurrence of physical morbidity, both at the time ICU discharge, and after hospital discharge. We observed delayed cuff deflation to be associated with prolonged MV, ICU LOS, and incidence of VAP and ICU mortality. This finding supports a possible association between earlier cuff deflation and VAP prevention, as seen in other studies [14]. However, our failure to adopt a validated VAP diagnostic criterion to clinical cases raises concerns around misclassification of VAP; therefore, this finding must be interpreted cautiously.

In line with other published data, we observed high rates of ICU-AW, delirium, anxiety and depression in this complex cohort. Interestingly, we noted significantly higher levels of anxiety and psychological distress in patients at ICU discharge where cuff deflation and, hence, voice restoration were delayed. It could be hypothesised this may be related to improved communication with care providers and relatives where cuff deflation is achieved earlier. Interestingly, this perceived benefit to communication did not translate into a reduction in delirium incidence in those with earlier cuff deflation. However, our adoption of a binary outcome of ‘having or not having experienced an episode of delirium’ may have been insensitive to any influence of cuff deflation, particularly since the incidence of delirium was relatively high, compared to previous literature. In future studies, alternative measures of delirium such as the number of days spent delirious should be considered. However, given the significant financial and rehabilitation burden WIND 3 patients place on ICUs, this positive trend towards improved clinical and patient outcomes with early cuff deflation warrants further investigation in more robust, prospective clinical trials.

### Limitations

Our study has several limitations, which must be considered when interpreting the findings. Primarily, the single-site design means that results may not be generalisable to other sites, particularly those with different case mixes or treatment protocols. Secondly, the lack of a standardised protocol around cuff deflation timing during the study period meant that practice was at the discretion of the treating clinician; hence, this would have been influenced by their experience and beliefs, and potentially subject to both conscious and unconscious bias. Thirdly, whilst baseline patient characteristics were generally similar across the subgroups of cuff deflation timing, it is possible that other unmeasured or intangible differences existed. As such, significant associations between the timing of cuff deflation and patient outcomes may have been subject to confounding by other factors and, hence, cannot be assumed to represent causal effects. Fourthly, the rates and timing of many of the outcomes and weaning milestones were undefined for those patients who either died or were transferred from the ICU prior to these being achieved. As such, analyses of these outcomes are only generalisable to the subgroup of patients for whom data were recorded, which may represent a biased subset of the cohort. Fifthly, data for safety concerns and adverse events related to cuff deflation could not be reliably collected retrospectively; hence, potential associations between these and the timing of cuff deflation could not be assessed. Finally, in the absence of a standardised criteria for VAP, this was identified based on the consultant-led decision to commence the appropriate antibiotics, which may have resulted in misclassification, potentially resulting in an overestimate of the prevalence.

In summary, many of these limitations were a result of the retrospective single-site nature of the study. As such, future studies in this area should ideally be prospective and multi-site and should aim to collect data for a diverse range of validated and robust outcomes, including adverse events, which are relevant to the target population.

## Conclusion

This observational study provides valuable insights into the process and timing of tracheostomy cuff deflation in WIND 3 patients. Additionally, we have described in detail the physical, cognitive and psychological outcomes associated with prolonged tracheostomy weaning. We present a potential and plausible association between delayed tracheostomy cuff deflation and several adverse outcomes. Delays in cuff deflation may be in part causal of, rather than consequent to, prolonged weaning and associated physical and psychological morbidity. Our results provide the foundations for future high-quality, prospective trials on the benefits and risks associated with early tracheostomy cuff deflation in patients requiring prolonged MV.

## References

[1] Abe T, Madotto F, Pham T, Nagata I, Uchida M, Tamiya N, et al. Epidemiology and patterns of tracheostomy practice in patients with acute respiratory distress syndrome in ICUs across 50 countries. Crit Care. 2018;22(1):195. doi: 10.1186/s13054-018-2126-6 30115127 PMC6097245

[2] Boles J-M, Bion J, Connors A, Herridge M, Marsh B, Melot C, et al. Weaning from mechanical ventilation. Eur Respir J. 2007;29(5):1033–56. doi: 10.1183/09031936.00010206 17470624

[3] Dolinay T, Hsu L, Maller A, Walsh BC, Szűcs A, Jerng J-S, et al. Ventilator weaning in prolonged mechanical ventilation-a narrative review. J Clin Med. 2024;13(7):1909. doi: 10.3390/jcm13071909 38610674 PMC11012923

[4] Freeman BD. Tracheostomy Update: When and How. Crit Care Clin. 2017;33(2):311–22. doi: 10.1016/j.ccc.2016.12.007 28284297

[5] Cinotti R, Voicu S, Jaber S, Chousterman B, Paugam-Burtz C, Oueslati H, et al. Tracheostomy and long-term mortality in ICU patients undergoing prolonged mechanical ventilation. PLoS One. 2019;14(10):e0220399. doi: 10.1371/journal.pone.0220399 31577804 PMC6774500

[6] Khalaila R, Zbidat W, Anwar K, Bayya A, Linton DM, Sviri S. Communication difficulties and psychoemotional distress in patients receiving mechanical ventilation. Am J Crit Care. 2011;20(6):470–9. doi: 10.4037/ajcc2011989 22045144

[7] McGrath B, Lynch J, Wilson M, Nicholson L, Wallace S. Above cuff vocalisation: A novel technique for communication in the ventilator-dependent tracheostomy patient. J Intensive Care Soc. 2016;17(1):19–26. doi: 10.1177/1751143715607549 28979454 PMC5606385

[8] Romero CM, Marambio A, Larrondo J, Walker K, Lira M-T, Tobar E, et al. Swallowing dysfunction in nonneurologic critically ill patients who require percutaneous dilatational tracheostomy. Chest. 2010;137(6):1278–82. doi: 10.1378/chest.09-2792 20299629

[9] De Leyn P, Bedert L, Delcroix M, Depuydt P, Lauwers G, Sokolov Y, et al. Tracheotomy: clinical review and guidelines. Eur J Cardiothorac Surg. 2007;32(3):412–21. doi: 10.1016/j.ejcts.2007.05.018 17588767

[10] Whitmore KA, Townsend SC, Laupland KB. Management of tracheostomies in the intensive care unit: a scoping review. BMJ Open Respir Res. 2020;7(1):e000651. doi: 10.1136/bmjresp-2020-000651 32723731 PMC7390235

[11] Rose L, Messer B. Prolonged Mechanical Ventilation, Weaning, and the Role of Tracheostomy. Crit Care Clin. 2024;40(2):409–27. doi: 10.1016/j.ccc.2024.01.008 38432703

[12] Pierson DJ. Tracheostomy and weaning. Respir Care. 2005;50(4):526–33. doi: 10.4187/respcare.05500526 15807916

[13] Freeman-Sanderson AL, Togher L, Elkins MR, Phipps PR. Return of Voice for Ventilated Tracheostomy Patients in ICU: A Randomized Controlled Trial of Early-Targeted Intervention. Crit Care Med. 2016;44(6):1075–81. doi: 10.1097/CCM.0000000000001610 26855430

[14] Blackwood B, Burns KEA, Cardwell CR, O’Halloran P. Protocolized versus non-protocolized weaning for reducing the duration of mechanical ventilation in critically ill adult patients. Cochrane Database Syst Rev. 2014;2014(11):CD006904. doi: 10.1002/14651858.CD006904.pub3 25375085 PMC6517015

[15] Dolinay T, Hsu L, Maller A, Walsh BC, Szűcs A, Jerng J-S, et al. Ventilator weaning in prolonged mechanical ventilation-a narrative review. J Clin Med. 2024;13(7):1909. doi: 10.3390/jcm13071909 38610674 PMC11012923

[16] Hernandez G, Pedrosa A, Ortiz R, Cruz Accuaroni M del M, Cuena R, Vaquero Collado C, et al. The effects of increasing effective airway diameter on weaning from mechanical ventilation in tracheostomized patients: a randomized controlled trial. Intensive Care Med. 2013;39(6):1063–70. doi: 10.1007/s00134-013-2870-7 23471512

[17] National Institute for Health and Clinical Excellence. Clinical Guideline CG83 – Rehabilitation after Critical Illness in Adults. 2009. https://www.nice.org.uk/guidance/cg83

[18] Intensive Care Society. Guidelines for the provision of intensive care services. Intensive Care Society. 2022. https://www.ics.ac.uk/GPICS_V2.1

[19] Béduneau G, Pham T, Schortgen F, Piquilloud L, Zogheib E, Jonas M, et al. Epidemiology of weaning outcome according to a new definition. The WIND Study. Am J Respir Crit Care Med. 2017;195(6):772–83. doi: 10.1164/rccm.201602-0320OC 27626706

